# Profiling of Individual Desulfo-Glucosinolate Content in Cabbage Head (*Brassica oleracea* var. *capitata*) Germplasm

**DOI:** 10.3390/molecules25081860

**Published:** 2020-04-17

**Authors:** Shiva Ram Bhandari, Juhee Rhee, Chang Sun Choi, Jung Su Jo, Yu Kyeong Shin, Jun Gu Lee

**Affiliations:** 1 Department of Horticulture, College of Agriculture & Life Sciences, Jeonbuk National University, Jeonju 54896, Korea; shivarbhandari@gmail.com (S.R.B.); jjs446@naver.com (J.S.J.); milkyway_100@naver.com (Y.K.S.); 2 National Agrobiodiversity Center, National Institute of Agricultural Sciences, Rural Development Administration, Jeonju 54874, Korea; rheehk@korea.kr; 3 Breeding Research Institute, Koregon Co., Ltd., Gimje 54324, Korea; sunlog@naver.com; 4 Institute of Agricultural Science & Technology, Jeonbuk National University, Jeonju 54896, Korea

**Keywords:** cabbage, genotypic variation, glucobrassicin, glucoiberin, glucosinolates, HPLC

## Abstract

Individual glucosinolates (GSLs) were assessed to select cabbage genotypes for a potential breeding program. One hundred forty-six cabbage genotypes from different origins were grown in an open field from March to June 2019; the cabbage heads were used for GSL analyses. Seven aliphatics [glucoiberin (GIB), progoitrin (PRO), epi-progoitrin (EPI), sinigrin (SIN), glucoraphanin (GRA), glucoerucin (GER) and gluconapin (GNA)], one aromatic [gluconasturtiin (GNS)] and four indolyl GSLs [glucobrassicin (GBS), 4-hydroxyglucobrassicin (4HGBS), 4-methoxyglucobrassicin (4MGBS), neoglucobrassicin (NGBS)] were found this study. Significant variation was observed in the individual GSL content and in each class of GSLs among the cabbage genotypes. Aliphatic GSLs were predominant (58.5%) among the total GSLs, followed by indolyl GSL (40.7%) and aromatic GSLs (0.8%), showing 46.4, 51.2 and 137.8% coefficients of variation, respectively. GIB, GBS and NGBS were the most common GSLs found in all genotypes. GBS was the most dominant GSL, with an average value of 3.91 µmol g^−1^ (0.79 to 13.14 µmol g^−1^). SIN, GIB, PRO and GRA were the other major GSLs, showing average values of 3.45, 1.50, 0.77 and 0.62 µmol g^−1^, respectively. The genotypes with relatively high contents of GBS, SIN, GIB and GRA warrant detailed studies for future breeding programs since the hydrolysis products of these GSLs have several anti-cancer properties.

## 1. Introduction

Glucosinolates (GSLs), sulfur-containing compounds, are exclusively found in order Brassicales. They are derived from the amino acid biosynthetic pathway and are associated to the characteristic pungent flavor and odor of *Brassica* vegetables. GSLs are enzymatically hydrolyzed to isothiocyanates (ITCs), thiocyanates or nitriles by the endogenous enzyme myrosinase depending upon the nature of the GSLs [1,2]. To date, about 132 different GSLs have been identified and characterized in a range of *Brassica* which have a specific GSL profile and content [3,4]. GSLs are classified into aliphatic, aromatic and indolyl compounds based on the structure of their side chain and the type of the precursor amino acid [2,5,6]. The GSLs and their breakdown products are known to have biologic and pharmacological effects, such as anti-fungicidal, anti-oxidative, anti-bacterial and anti-cancer [7,8,9,10,11,12]. The isothiocyanates (sulforaphane, iberin, phenylethyl and prop-2-enyl) derived from glucoraphanin, glucoiberin, gluconasturtiin and sinigrin, respectively, have anti-proliferative and anti-cancer properties [13,14,15]. Furthermore, degradation products such as indole-3-carbinol (I3C) and 3,3′-diindolylemethane (DIM) from an indolyl GSL; glucobrassicin also showed the activation of cancer preventive enzyme after hydrolysis [7]. Phenylethyl isothiocyanate hydrolyzed from gluconastrutiin shows antimicrobial [13] and anti-cancer activity [10,16] against prostate and colon cancer by apoptosis. The alternative use of GSLs as synthetic pesticides for pest and disease control and bionematicides is also reported [17,18]. Some aliphatic GSLs including sinigrin and progoitrin are also responsible for the bitter flavor of the *Brassica* vegetables and may influence the consumer acceptance [2,19]. Therefore, the GSLs in *Brassica* vegetables should be investigated due to their dietary and medicinal properties.

Among the green *Brassica* vegetables cultivated globally, cabbage (*Brassica oleracea* var. *capitata*) is one of the commonly grown and consumed due to its low cost and health promoting properties. It is highly nutritious and possess a wide range of health-promoting bioactive compounds such as GSLs, vitamins, phenols, anthocyanin and carotenoids [12,20,21,22,23,24,25]. The profile and content of given bioactive compounds varies due to genetic and environmental factors such as growth seasons and conditions and developmental stages [20,21,22,24,26,27,28]. GSL profile and their content have been studied in several *Brassica* vegetables: broccoli [29], radish [30], Chinese cabbage [31], rape [32], turnip [33,34] and pakchoi [35]. Furthermore, studies have been undertaken to understand how the GSL profiles and content in cabbage is affected by genotypes [20,21,22], growth seasons [20,36], developmental stages of different tissues [25,26,37], and postharvest storage [38,39]. However, most of the studies related to the GSLs analysis in cabbage have been limited to a small number of genotypes as well as to the effects of the environmental factors [20,21,22]. Furthermore, there is currently no information regarding variation in GSL profiles and their content using a large number of the genetic resources of different origin. Therefore, in this study we aimed to analyze the GSL profile and content in the heads of 146 cabbage genotypes grown in open field with identical conditions to investigate genetic variations and then to select the candidate genotype based on the targeted GSLs that may be used for the generation of high-GSLs containing genotypes.

## 2. Results and Discussion

### 2.1. Variation in Agronomic Characteristics

Qualitative and quantitative parameters indicating different agronomic traits were measured during and after the harvest. Qualitative parameters such as shape of the head and inner and outer color of the leaf showed different patterns (Table S1). The head of the most genotypes was round (124 genotypes) while the head shape of the remaining genotypes was flat, semi-flat, semi-round, round and pointed, semi-flat and round, semi-round, pointed and upper pointed and round. The outer and inner leaf color was green in 130 cabbage genotypes and red in the remaining 16 genotypes. The detailed information about the leaf color is presented in supplemental file. The quantitative parameters showed great variability among the genotypes (Table S1). Leaf length showed three-fold difference, ranging from 22 to 66 cm and the average of 39.9 cm. The average head width, head height and core length were 15.9, 15.1 and 6.4 cm, and showed 2.6-, 3.0- and 4.1-fold difference to the lower and higher value of each parameter, respectively. The weight of the cabbage head showed 14.7-fold difference, with a range from 190 to 2800 g.

### 2.2. Identification and Quantification of Individual GSLs Profile in Cabbage

**Figure 1:**
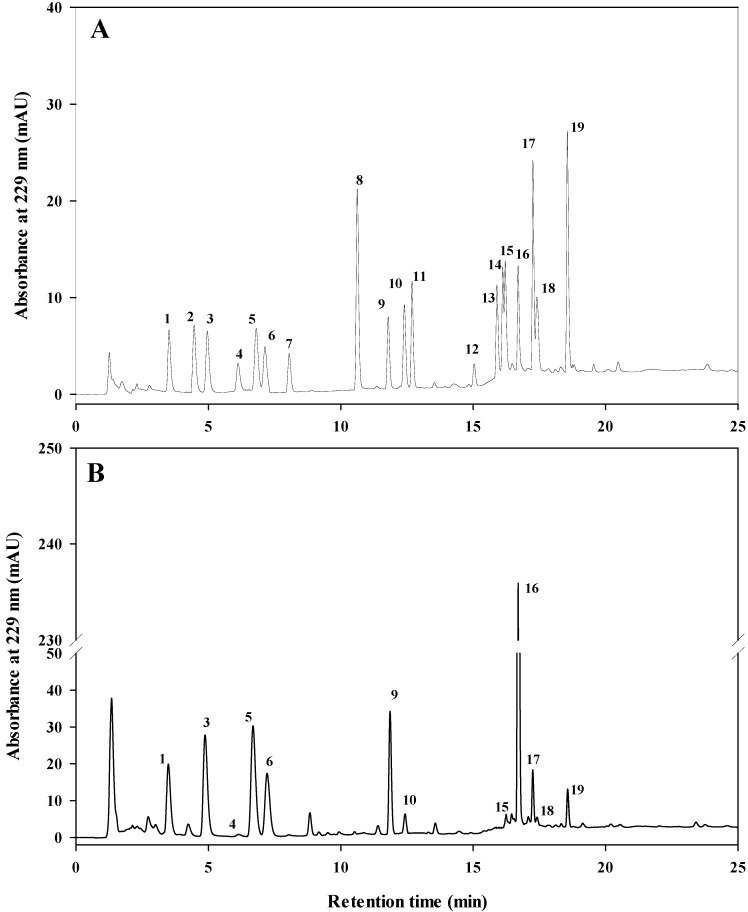
High performance liquid chromatography (HPLC) chromatogram of desulfo- glucosinolates (GSLs) in standard mixture (**A**) and cabbage sample (**B**). Refer to Table 1 for peak identification.

Among the analyzed 19 GSLs, twelve GSLs were identified in both green and red cabbage genotypes and quantified by using HPLC as presented in Figure 1 and Table 1. Seven aliphatic, one aromatic and four indolyl GSLs were identified based on the retention time of HPLC chromatograms. Each of the GSL clearly separated in both the standard mixture and cabbage sample. The detected GSLs in cabbage genotypes were GIB, PRO, EPI, SIN, GRA, GNA, GER, GBS, 4HGBS, 4MGBS, NGBS and GNS (Figure 1B) mostly consistent with previous findings [20,22,40]. The identified GSLs were quantified using the standard curves created from commercial standards (Table 1).

### 2.3. Variation in Individual and Total GSL Content among 146 Cabbage Genotypes

**Figure 2:**
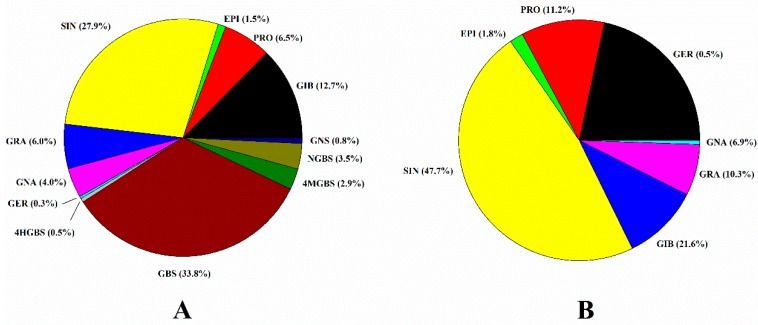
Individual GSL content (%) in total GSLs (**A**) and aliphatic GSLs (**B**) in 146 cabbage genotypes.

The total glucosinolate content varied from 3.99 to 23.75 μmol g^−1^ DW (Table 2) and was significantly different between the genotypes. The aliphatic GSLs were predominant, representing the 58.5% of the total GSLs, followed by indolyl GSL (40.7%) and aromatic GSLs (0.8%). The average content of aliphatic, indolyl and aromatic GSLs were 6.95, 4.46 and 0.09 μmol g^−1^ DW with an over 46.4%, 51.2% and 137.8% coefficient of variation, respectively. Among the individual GSLs, GBS was the most dominant comprising 33.8% of the total GSLs (Figure 2A), followed by SIN (27.9%), GIB (12.7%), PRO (6.5%) and GRA (6.0%). SIN represented the major aliphatic GSL comprising of an average of 47.7%, followed by GIB (21.6%), PRO (11.2%) and GRA (10.3%) (Figure 2B). The remaining aliphatic GSLs—GNA, EPI and GER comprised 6.9%, 1.8% and 0.5%, respectively.

**Figure 3:**
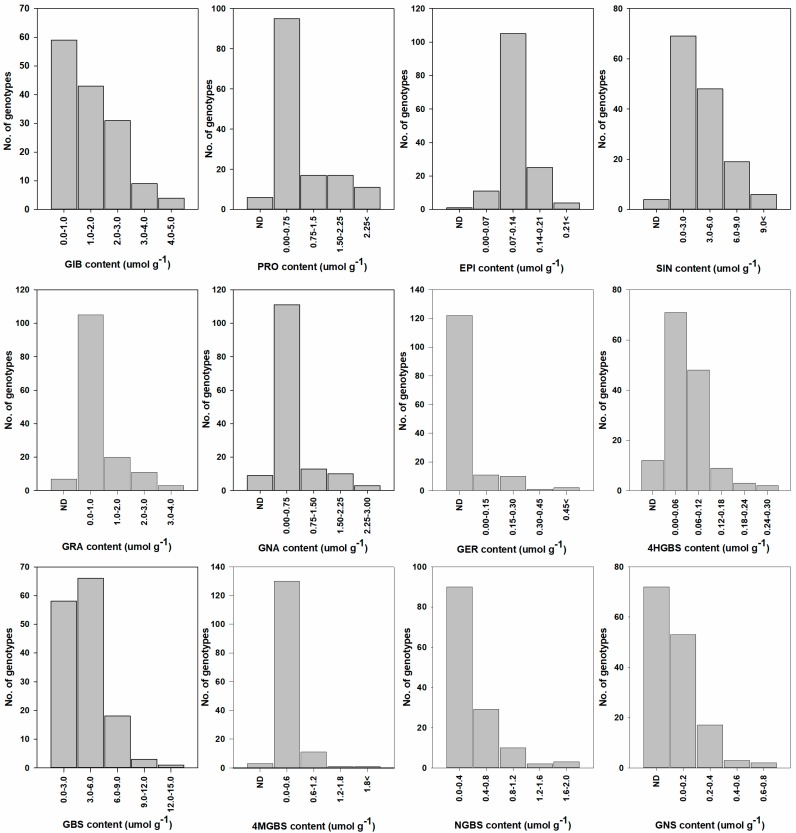
Frequency of individual GSL distribution among the 146 cabbage genotypes. GIB: glucoiberin; PRO: progoitrin; EPI: epiprogoitrin; SIN: sinigrin; GRA: glucoraphanin; GNA: gluconapin; GER: glucoerucin; 4HGBS:4-hydroxyglucobrassicin; GBS: glucobrassicin; 4MGBS: 4-methoxyglucobrassicin; NGBS: neoglucobrassicin; GNS: gluconastrutiin; ND: not detected.

GIB, GBS and NGBS were found in all the genotypes among the detected 12 GSLs (Figure 3, Table S2). GBS was the most dominant GSL in 77 genotypes (Table S2) which account for 53% of the total genotypes and ranged from 0.79 to 13.14 μmol g^−1^ DW with an average of 3.91 μmol g^−1^ DW and exhibited 53.5% variation coefficient (Table 2). Among the 146 genotypes, 124 genotypes showed relatively low GBS content (<6.0 μmol g^−1^ DW) while 18 genotypes showed medium (6.0–9.0 μmol/g DW) and the remaining four genotypes showed the highest (> 9.0 μmol g^−1^ DW) GBS content (Figure 3). Our results are similar to Choi et al. [20] and Cartea et al. [40] who also found the GBS to be the most dominant GSL. However, the GBS content found in our study is significantly higher to those reported by Park et al. [21] who found that GBS content varied between cabbage varieties from 0.1 to 8.0 μmol g^−1^ DW. The higher GBS content found in our study may be due to the larger number of cabbage genotypes and the diversity of their origins.

SIN was the most dominant GSL in 62 genotypes accounting 42% of the total genotypes and its content ranged from 0.01 to 12.87 μmol g^−1^ DW with the average value of 3.55 μmol g^−1^ DW (Table 2). Most of the genotypes (117 genotypes) showed the range between 0.01 to 6.0 μmol g^−1^ DW (Figure 3). SIN content was higher than 6.0 μmol g^−1^ DW in 25 genotypes, consistent with Park et al. [21] who also found SIN to be the most dominant GSLs and within the range of this study. Likewise, Pocock et al. [41] found higher levels of SIN and GBS compared to the other GSLs in cabbage. The presence of high SIN in cabbage genotypes implies the pharmacological value as it shows the anti-glycation activity [42], inhibition of the proliferation of tumor cells and adipocyte differentiation [43], antioxidant activity [44] and suppressive effects towards hypertriglyceridemia [45]. The third most dominant GSL, GIB was found in all the genotypes ranging from 0.01 to 5.01 μmol g^−1^ DW with an average of 1.51 μmol g^−1^ DW. GIB comprised the 12.7% and 21.6% of the total GSLs and aliphatic GSLs (Figure 2). GIB content was the lowest (<1.0 μmol g^−1^ DW) in 59 genotypes, followed by medium levels (1.0–2.0 μmol g^−1^ DW) detected in 43 genotypes and the highest GIB content (4.0–5.0 μmol g^−1^ DW) detected in four genotypes (Figure 3). The value reported herein is relatively lower compared to the previous reports by Cartea et al. [40] who found 2.7–12.1 μmol g^−1^ GIB in 26 cabbage varieties grown in Spain. Such discrepancies may be due to the difference in genotypes as well as environmental conditions. Furthermore, the GIB and SIN content in the cabbage genotypes was relatively higher than in broccoli [29] emphasizing its higher health beneficial value. The high content of SIN, GIB and GBS in cabbage genotypes indicates a high pharmacological value of this crop as their breakdown products are allyl isothiocyanate, iberin and indol 3-carbinol, respectively [7] and the hydrolysis products of these compounds have several anticancer properties [46,47,48].

GRA, which is considered as the most important GSL due to its anticancer activities, was found in most of the genotypes (Figure 3). Many researchers are interested in enhancing the GRA content in *Brassica* vegetables due to its health-promoting activities [15]. Similar to Park et al. [21], GRA was the second most dominant GSLs among the aliphatic glucosinolates. However, we found a relatively low content (<1.0 μmol g^−1^ DW) of GRA in most genotypes (105 genotypes). GRA comprised 6.0% of the total GSLs and 10.3% of the aliphatic GSLs (Figure 2) with an average content of 0.62 μmol/g DW and 129.9% coefficient of variation among the genotypes. In contrast, Park et al. [21] found that GRA content varied from 5.29 to 14.91 μmol g^−1^ DW accounting to 44% of total GSLs and 77% of aliphatic GSLs in the 38 cabbage lines grown in fall season. These discrepancies may be due to the differences between the genotypes as well as growing conditions, as the level of GSLs varies significantly between genotypes and environmental conditions.

NGBS, the second most dominant indolyl GSLs, was found in all genotypes. NGBS content ranged from 0.1 to 2.0 μmol g^−1^ DW with an average of 0.4 μmol g^−1^ DW, however, most of the genotypes had less than 1.2 μmol/g DW of NGBS. Our result showed relatively higher NGBS compared to the previous reports by Park et al. [21] in 45 lines of green and red cabbage. PRO, which is responsible for the bitter flavor and influences consumer choice [2], was also present in most of the genotypes, however most the genotypes showed >2.00 μmol g^−1^ DW and the coefficient of variation was 106%. The other GSLs constituted a small portion of the total GSL content: on average, 1.5% for EPI, 4.0% for GNA, 0.5% for 4HGBS, 0.3% for GER, 2.9% for 4MGBS and 0.8% for GNS. Cartea et al. [40] also found similar results with the lower content of these GSLs while analyzing 26 cabbage varieties. Among the detected GSLs, GER was observed in the lowest number of genotypes (~16%) and its content was less than 0.60 μmol g^−1^ DW. Among the 12 quantified GSLs in cabbage, GER showed the highest coefficient of variation (280.5%), which was followed by GNS (137.8%) and GRA (129.9%), whereas EPI exhibited the lowest genetic variation (36.1%) (Table 2).

Overall, the differences in total and individual GSL content were observed among the genotypes. The degree of genotypic variation was different for the individual GSL. The results found in this study are similar with previous reports in different *Brassica* vegetables including cabbage [20,21,22,29,31,33]. However, this study provides more information about the GSL profile in a large number of cabbage varieties. The variation in GSLs levels found in this study implied that the potential health benefit of the cabbage crop is greatly influenced and dependent on the genotype.

### 2.4. Selection of Candidate Genotypes for the Breeding Program

**Figure 4:**
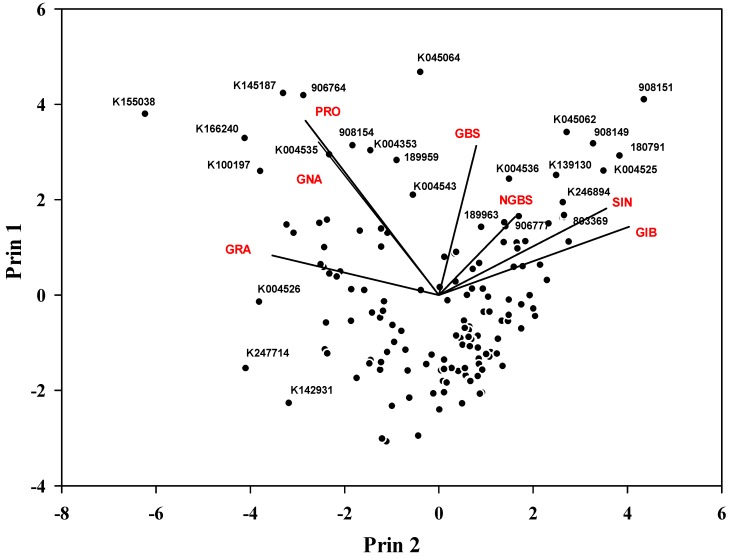
Principal component analysis (PCA) of individual major GSLs. GRA: glucoraphanin; GNA: gluconapin; PRO: progoitrin; GBS: glucobrassicin; NGBS: neoglucobrassicin; SIN: sinigrin; GIB: glucoiberin.

After analyzing the individual GSLs and their content, some of the genotypes were selected based on the targeted GSLs (Table 3). Specifically, genotypes with the highest GBS, IBE, GRA and SIN content may have a potential for health benefits, as the hydrolysis product of these GSLs show anticancer properties [7,9,28,46,49]. PCA for individual GSLs showed that some selected genotypes had higher specific GSL content (Table 3 and Figure 4). GBS was the highest in 908151, 906777, 189963 and 180,791 genotypes. Similarly, four genotypes, namely K004525, 180791, 803374 and 803360, showed a relatively higher GIB content than other GSLs. Six genotypes—908149, K045062, K246894, K139130, 907279 and 803369—had a relatively higher SIN content (>9.0 μmol g^−1^ DW) than the other genotypes; it was the highest in 908149 genotype. PCA further revealed that three genotypes—803346, 803372 and K004527—had a higher GIB content (>4.0 μmol g^−1^ DW) than the other genotypes. Three genotypes (K142931, K004526 and K247741) had a higher GRA content (>3.0 μmol g^−1^ DW), although total GSL content was lower compared to the other selected genotypes. These results suggest the use of these specific genotypes in breeding program to increase GSL content. Furthermore, five genotypes exhibited a higher PRO content, which should be considered given that high PRO content may affect the consumer choice [2] as it produces oxazolidine-2-thione, which causes goiters in mammals and other harmful effects [19,50]. To the best of our knowledge, this is the first report reporting GSLs variation in a large number of cabbage genotypes with accurate quantification using 19 authentic standards in HPLC as most of the other previous studies were based on the response factors or LC-MS-based.

### 2.5. Correlation Analysis among the GSLs

The correlation analysis was performed to investigate the accumulation pattern of individual GSLs and their interactions. In this study, individual GSLs correlated to each other differently (Table 4). The results showed a high significant correlation between major GSLs. The highest positive correlation was found between PRO and GNA (r = 0.805**) this is because PRO is directly synthesized from GNA [51]. On the other hand, SIN exhibited either significantly negative or non- significant correlation with GRA (r = −0.555*), GER (r = −0.271**), PRO (r = −0.106) and EPI (r = −0.065) which was probably due to the difference in their intermediates biosynthetic pathway [51]. GIB showed significant correlation with almost all of the GSLs with the highest positive correlation with SIN which may be because of the same biosynthetic pathway between the GIB and SIN [52]. Among the indolyl GSLs, 4HGBS showed statistically insignificant correlation with almost all of the aliphatic and indolyl GSLs which may be due to the lower content of 4MGBS in almost all of the genotypes although it has the same precursor; tryptophan, for the biosynthesis of indolyl GSLs [51]. The most dominant GSL, GBS showed either significant positive or statistically insignificant correlation to the other GSLs with the highest positive correlation with NGBS (r = 0.411**). Similar to the previous reports by Bhandari et al. [26], the aliphatic GSLs, namely PRO, GNA, GRA and GER, showed a strongly positive correlation with each other as these GSLs are 4-carbon aliphatic GSLs and follow a similar biosynthetic pathway [6,51]. The total GSL content had the highest significantly positive correlation with SIN (r = 0.658**), followed by GBS (r = 0.564**) and GIB (r = 0.576**) as these GSLs have high contribution to the total GSL content in cabbage.

## 3. Materials and Methods

### 3.1. Chemicals and Reagents

Nineteen GSL standards, namely glucoiberin (GIB), glucolepidiin (GLP), progoitrin (PRO), epiprogoitrin (EPI), glucoraphanin (GRA), glucoraphenin (GRE), sinigrin (SIN), gluconapin (GNA), sinalbin (SNB), glucomoringin (GMR), glucobarbarin (GBA), glucotropaeolin (GTR), glucobrassicanapin (GBN), glucoerucin (GER), glucobrassicin (GBS), 4-hydroxyglucobrassicin (4HGBS), 4-methoxyglucobrassicin (4MGBS), neoglucobrassicin (NGBS) and gluconasturtiin (GNS), were purchased from Cfm Oskar Co. (Marktredwitz, Germany). Diethylaminoethyl (DEAE)- Sephadex-A25, sodium acetate, HCl, and aryl sulfatase (EC 3.1.6.1, type H-1) from *Helix pomatia*, were obtained from Sigma-Aldrich (St. Louis, MO, USA). HPLC grade acetonitrile, methanol, and water were obtained from Avantor Performance Materials (Center Valley, PA, USA).

### 3.2. Plant Material and Cultivation

One hundred forty-six cabbage genotypes (16 red and 130 green cabbage) collected from different countries were used in this study. Individual names and source details are presented in Table S1. The seeds were obtained from National Agrobiodiversity Center, Jeonju, South Korea. The seeds were sown in 72-cell trays on March 10, 2019 and grown in a nursery until April 10, 2019 at the Breeding Research Institute of Koregon (Gimje, South Korea). The seedlings (30 days after sowing) were then transplanted into the experimental field in rows with 30 cm between plants and 100 cm between rows. The experimental field contained the base fertilizer: N (117 kg ha^−1^), P (72.5 kg ha^−1^), K (72.5 kg ha^−1^), M (18 kg ha^−1^) and B (1.8 kg ha^−1^). Irrigation was performed daily (in the morning) using sprinklers. Cabbage heads were harvested at maturity, 40–100 days after transplanting depending on the genotype. During the harvest, leaf length was measured. After harvest, cabbage heads were immediately brought to the laboratory and 2–3 outer leaves were removed due to the dust particles. Then, the outer and inner leaf color, head shape, height, width, weight and core length were measured and the head height to width ratio was calculated. Each cabbage head was sliced vertically into four parts with a knife, cut into small pieces, freeze-dried at −54 °C and stored at −20 °C until GSL analysis.

### 3.3. Extraction of Intact Glucosinolates (GSLs) and Their Desulfation

The GSLs extraction procedure was performed according to the method described by Bhandari et al. [26]. Briefly, freeze-dried and powdered cabbage samples (0.05 g) were extracted twice with 1 mL of boiling methanol (70%) for 20 min to deactivate the myrosinase. After centrifugation (12,000 × g, 10 min, 4 °C), the supernatant was transferred to a 2-mL tube. The supernatant from the two extractions was combined and considered as crude GSLs. The crude GSLs extract was added into a Mini Bio-Spin Chromatography Column (Bio-Rad Laboratories, Hercules, CA, USA), filled with 0.5 mL diethylaminoethyl (DEAE)-Sephadex A-25 (Sigma Aldrich, St. Louis, MO, USA), which had been activated with 0.1-M sodium acetate (pH 4.0). Then, 200 µL of purified aryl sulfatase (EC 3.1.6.1, type H-1 from *H. pomatia*; Sigma-Aldrich) was added, the column was capped from both sides and left for 18 h at room temperature for the desulfation of GSLs. The desulfo-GSLs were diluted with 3 × 0.5 mL distilled water, filtered through a 0.2-µm PVDF syringe filter and analyzed immediately using HPLC.

### 3.4. Separation and Identification of Individual GSLs Using High Performance Liquid Chromatography (HPLC)

Desulfo-GSLs were analyzed using 1260 HPLC system (Agilent Technologies, Santa Clara, CA, USA) equipped with auto-injector and a photodiode array (PDA) detector set at 229 nm. The Acquity UPLC ^®^BEH-C18 Column (1.7 μm, 2.1 × 100 mm; Waters Co., Milford, MA, USA) protected by a guard column was used for the separation of GSLs at a column oven temperature of 30 °C. The mobile phase was ultrapure water (A) and 100% acetonitrile (B) at a flow rate of 0.2 μL min^−1^ with a gradient program as: a linear step from 0.5% to 10.0% of solvent B within 10 min, followed by linear up to 30% in 20 min, 0.5% of solvent B at 21 min and isocratic conditions with 0.5% of solvent B till 30 min. The individual commercial GSLs were identified and quantified with the authentic standards with their retention time and HPLC area, respectively. All the GSLs standards were desulfated in the same way as the sample preparation. Varying concentration of each GSL standard (1–20 μL mL^−1^) was used to create the standard curve. All samples were analyzed in triplicates and the individual desulfo-GSLs was expressed as µmol g^−1^ in dry weight (DW). All the experimental results were designated as GSLs although desulfo-GSLs were determined in this study.

### 3.5. Statistical Analyses

The results are reported as mean of three replications. A principal component analysis (PCA) was performed to calculate the effect of genotypes on the GSL profile. Relationship among individual GSLs was computed using Pearson’s correlation coefficient (r) at *P* < 0.05 using SPSS version 20 (IBM, Armonk, NY, USA). All figures were computed by using SigmaPlot 12^®^.

## 4. Conclusions

The variation in the GSL content and their profile reported in this study suggests that the potential health benefits of cabbage are greatly dependent on the genotype. This study provided valuable information about the GSL content and profile in both green and red cabbage genotypes. Furthermore, the content of each GSL was differently affected by genotype showing different magnitude of variation. The analysis of individual GSLs in cabbage lines would be useful for choosing the best inbred lines. The presence of GIB, SIN, GRA and GBS in different genotypes should be studied in more detail as these GSLs are the precursors of isothiocyanates with anti-cancer properties. The findings in this study could be used for developing new lines of cabbage having specific GSL profile and content.

## Figures and Tables

**Table 1:** High performance liquid chromatography (HPLC) determination of 19 desulfo-glucosinolates studied in cabbage genotypes.

SN	Retention Time (min)	Common Name	Semisystematic Name of R-Group	Abbreviation	Group	Linearity Curve	R^2^
1	3.51	Glucoiberin	3-Methylsulfinylpropyl	GIB	Aliphatic	y = 30.468x + 0.236	0.9999
2	4.46	Glucolepidiin	Ethyl	GLP	Aliphatic	y = 38.502x − 0.447	0.9999
3	4.95	Progoitrin	2-Hydroxy-3-butenyl	PRO	Aliphatic	y = 35.060x − 0.647	0.9999
4	6.11	Epiprogoitrin	(2R)-2-Hydroxy-3-butenyl	EPI	Aliphatic	y = 18.618x − 0.537	0.9999
5	6.81	Sinigrin	2-Propenyl	SIN	Aliphatic	y = 43.343x − 0.236	0.9999
6	7.15	Glucoraphanin	4-Methylsulfinylbutyl	GRA	Aliphatic	y = 31.056x + 2.932	0.9992
7	8.06	Glucoraphenin	4-(Methylthio)butyl	GRE	Aliphatic	y = 7.5889x − 2.068	0.9989
8	10.62	Sinalbin	4-hydroxybenzyl	SNB	Aromatic	y = 132.13x − 6.977	0.9999
9	11.78	Gluconapin	3-Butenyl	GNA	Aliphatic	y = 30.566x − 0.487	0.9999
10	12.34	4-Hydroxyglucobrassicin	4-Hydroxy-3-indolylmethyl	4HGBS	Indolyl	y = 53.070x − 1.559	0.9996
11	12.67	Glucomoringin	4-(α-l-rhamnopyranosyloxy)benzyl	GMR	Aromatic	y = 9.494x − 0.769	0.9990
12	15.01	Glucobarbarin	2-Hydroxy-2-phenylethyl	GBA	Aliphatic	y = 35.957x + 0.660	0.9997
13	15.89	Glucobrassicanapin	4-Pentenyl	GBN	Aliphatic	y = 34.109x − 0.258	0.9999
14	16.11	Glucotropaeolin	Benzyl	GTR	Aromatic	y = 34.081x + 0.067	0.9999
15	16.22	Glucoerucin	4-(Methylthio)butyl	GER	Aliphatic	y = 29.598x − 5.741	0.9994
16	16.67	Glucobrassicin	3-Indolylmethyl	GBS	Indolyl	y = 67.591x + 0.541	0.9997
17	17.24	4-Methoxyglucobrassicin	4-Methoxy-3-indolylmethyl	4MGBS	Indolyl	y = 81.427x +1.562	0.9996
18	17.42	Gluconasturtiin	2-Penylethyl	GNS	Aromatic	y = 20.730x − 0.538	0.9999
19	18.56	Neoglucobrassicin	N-Methoxy-3-indolylmethyl	GNBS	Indolyl	y = 90.766x − 1.228	0.9997

**Table 2:** The variation of glucosinolates in cabbage heads from 146 genotypes.

GSLs ^a^	Average (μmol g^−1^)	Range (μmol g^−1^)	CV (%) ^b^
GIB	1.50	0.01–5.01	69.93
PRO	0.77	0.00–3.86	106.57
EPI	0.11	0.00–0.26	36.13
SIN	3.45	0.00–12.87	79.15
GRA	0.62	0.00–3.85	129.88
GNA	0.47	0.00–2.81	126.77
GER	0.03	0.00–0.61	280.47
Total Aliphatic GSLs	6.95	1.31–17.88	46.39
4HGBS	0.06	0.00–0.29	85.47
GBS	3.91	0.79–1.14	53.53
4MGBS	0.29	0.00–2.57	94.99
NGBS	0.40	0.05–2.00	84.33
Total Indolyl GSLs	4.46	1.01–15.57	51.16
GNS	0.09	0.00–0.67	137.77
Total Aromatic GSLs	0.09	0.00–0.67	137.77
Total	11.71	3.99–23.75	36.44

^a^ Glucosinolate abbreviations. GSLs: glucosinolates; GIB: glucoiberin; PRO: progoitrin; EPI: epiprogoitrin; SIN: sinigrin; GRA: glucoraphanin; GNA: gluconapin; GER: glucoerucin; 4HGBS:4-hydroxyglucobrassicin; GBS: glucobrassicin; 4MGBS: 4-methoxyglucobrassicin; NGBS: neoglucobrassicin; GNS: gluconastrutiin. ^b^ CV: coefficient of variation.

**Table 3:** Selected cabbage genotypes with the higher glucosinolate content.

IT No.	Temporary No.	Name	^a^Glucosinolate Content (μmol g^−1^ DW)												
			GIB	PRO	EPI	SIN	GRA	GNA	4HGBS	GER	GBS	4MGBS	GNS	NGBS	Total GSLs
^b^NA	908151	BOL-AWS-1999-156	3.86	0.20	0.11	3.46	0.20	0.16	0.03	^c^ND	13.14	0.21	0.67	1.73	23.76
NA	908149	BOL-AWS-1999-153	2.92	1.17	0.12	12.87	0.20	0.61	0.11	ND	4.09	0.26	0.29	0.74	23.37
NA	K045064	Valcatiecskaya	1.22	2.06	0.21	10.74	0.30	1.93	0.04	0.15	5.88	0.20	ND	0.58	23.31
180791	NA	Late Flat Dutch	4.87	0.49	0.09	5.77	0.31	0.23	0.11	ND	9.08	0.27	0.12	1.26	22.59
NA	K045062	Kashirka 202	3.16	0.90	0.12	6.88	0.54	0.46	0.28	ND	7.97	0.29	0.12	0.90	21.62
NA	K246894	Succession Green Leaved	2.83	0.25	0.14	9.53	0.09	0.05	0.06	ND	7.48	0.40	ND	0.49	21.33
NA	K139130	Sagyahwak	3.39	0.89	0.15	10.05	0.26	0.49	0.09	ND	4.90	0.17	0.24	0.10	20.72
NA	907279	Yujanka 31	2.79	1.03	0.09	11.29	0.20	0.53	0.06	ND	3.25	0.10	0.24	0.28	19.86
NA	K004525	Podarok	5.01	0.53	0.11	5.67	0.35	0.30	0.29	ND	5.87	0.37	0.16	0.81	19.49
NA	803369	Zuun kharaa No 10	2.35	0.52	0.10	10.29	0.13	0.56	0.04	ND	4.38	0.17	0.28	0.56	19.39
NA	803374	Zuun kharaa No 15	4.55	0.50	0.10	8.28	0.21	0.32	0.04	ND	4.51	0.39	ND	0.29	19.19
NA	K145187	Kirmizi	0.57	3.41	0.15	2.00	1.53	2.79	ND	0.16	6.40	0.22	ND	0.95	18.18
NA	906764	Natsuzoka	1.08	3.28	0.22	2.17	1.47	1.60	0.08	0.12	7.46	0.13	ND	0.22	17.82
NA	906777	UR Gogetsu	2.11	0.44	0.11	2.81	0.29	0.23	0.02	ND	10.60	0.47	0.26	0.33	17.66
NA	K004353	Rubin	1.00	3.28	0.17	2.56	2.04	ND	0.15	ND	7.76	0.19	ND	0.41	17.55
160677	NA	Gyeongphong 1 ho	3.15	0.86	0.10	6.33	0.31	0.25	0.17	ND	4.50	0.74	0.62	0.15	17.16
189963	NA	Skvirskaya N32	2.02	0.45	0.14	2.98	0.22	0.22	0.10	ND	10.10	0.39	ND	0.19	16.81
NA	K166205	153	1.84	0.08	0.09	4.11	0.06	0.08	0.02	ND	6.34	0.22	ND	1.96	14.78
NA	K155038	Tashkent 110	0.05	3.86	0.26	0.09	2.65	0.92	0.04	0.56	5.47	0.46	ND	0.36	14.71
NA	K166240	Red Drumhead 2	0.65	3.06	0.19	1.49	1.78	2.36	0.06	0.22	4.42	ND	ND	0.16	14.41
NA	803360	Zuun kharaa No 1	4.06	0.20	0.08	4.48	0.11	0.13	0.03	ND	4.03	0.17	ND	0.25	13.54
NA	K100197	Pourovo cervene	0.31	2.49	0.18	0.77	1.47	2.81	0.07	0.10	3.26	0.16	ND	0.42	12.03
204203	707561	Golden Acre	2.10	0.32	0.09	3.28	0.05	0.07	0.01	ND	3.48	0.11	0.10	1.70	11.32
NA	K142931	Sudya	0.01	0.01	0.04	0.01	3.36	0.03	0.06	0.26	4.03	2.55	ND	0.39	10.75
NA	K004526	Sudiya-146	0.05	1.62	0.11	0.03	3.85	0.52	0.07	0.18	2.51	1.09	0.22	0.15	10.40
NA	K247741	TJK-PHJ-2014-6-8	0.78	0.04	0.09	0.02	3.10	0.04	0.04	ND	3.72	0.69	0.27	0.11	8.90

^a^Glucosinolate abbreviation GIB: glucoiberin; PRO: progoitrin; EPI: epiprogoitrin; SIN: sinigrin; GRA: glucoraphanin; GNA: gluconapin; GER: glucoerucin; 4HGBS:4-hydroxyglucobrassicin; GBS: glucobrassicin; 4MGBS: 4-methoxyglucobrassicin; NGBS: neoglucobrassicin; GNS: gluconastrutiin. ^b^NA: Information not available, ^c^ND: Not detected.

**Table 4:** Correlation analysis among the GSLs analyzed in this study (n = 146).

GSLs	PRO	EPI	SIN	GRA	GNA	4HGBS	GER	GBS	4MGBS	GNS	NGBS	Total GSL
GIB	−0.285**	−0.131	0.598**	−0.485**	−0.273**	0.192*	−0.318**	0.239**	−0.112	0.272**	0.254**	0.576**
PRO		0.686**	−0.106	0.360**	0.805**	0.073	0.254**	0.099	−0.141	−0.031	−0.061	0.280**
EPI			−0.065	0.260**	0.592**	0.032	0.192*	0.231**	−0.195*	−0.001	−0.097	0.296**
SIN				−0.555**	−0.050	0.110	−0.271**	0.006	−0.180*	0.263**	0.113	0.658**
GRA					0.199*	−0.008	0.555**	0.193*	0.336**	−0.069	−0.010	−0.061
GNA						0.016	0.202*	−0.015	−0.183*	-0.052	−0.088	0.214**
4HGBS							−0.063	0.143	0.130	0.085	0.062	0.229**
GER								0.045	0.251**	−0.164*	−0.059	−0.019
GBS									0.089	0.132	0.411**	0.654**
4MGBS										−0.028	0.028	−0.017
GNS											0.110	0.308**
NGBS	□	□	□	□	□	□	□	□	□	□	□	0.393**

*,** Correlation is significant at *P* = 0.05 and 0.01, respectively. GSLs: glucosinolates; GIB: glucoiberin; PRO: progoitrin; EPI: epi-progoitrin; SIN: sinigrin; GRA: glucoraphanin; GNA: gluconapin; GER: glucoerucin; 4HGBS: 4-hydroxyglucobrassicin; GBS: glucobrassicin; 4MGBS: 4-methoxyglucobrassicin; NGBS: neoglucobrassicin; GNS: gluconastrutiin.

## Supplementary Materials

The following are available online in Table S1: The source detail and agronomic characteristic of cabbage genotypes (n = 146) and Table S2: Variation in GSL profile and concentration (µmol g^−1^ DW) in cabbage (n = 146).
