# Supplementary material for: Profiling of Individual Desulfo-Glucosinolate Content in Cabbage Head (Brassica oleracea var. capitata) Germplasm
Source: Molecules. 2020 Apr 17;25(8):1860. doi: 10.3390/molecules25081860 (PMC7221891; doi:10.3390/molecules25081860)
Supplement: Supplementary file 1 [file molecules-25-01860-s001.pdf]

Profiling of individual desulfo-glucosinolate content in cabbage head (*Brassica oleracea* var. *capitata*) germplasm

Shiva Ram Bhandari[1], Juhee Rhee[2], Chang Sun Choi[3], Jung Su Jo[1], Yu Kyeong Shin[1], and Jun Gu Lee[1,4,*]

[1] *Department of Horticulture, College of Agriculture & Life Sciences, Jeonbuk National University, Jeonju 54896, Korea*

[2]*National Agrobiodiversity Center, National Institute of Agricultural Sciences, Rural Development Administration, Jeonju* 54874, *Korea*

[3]*Breeding Research Institute, Koregon Co., Ltd, Gimje 54324, Korea*

[4]*Institute of Agricultural Science & Technology, Jeonbuk National University, Jeonju 54896, Korea*

***Corresponding author**: Jun Gu Lee, E-mail: jungu@jbnu.ac.kr

Tel: +82-63-270-2581

Supplementary Material

Table S1: The source detail and agronomic characteristic of cabbage genotypes (n=146)

SN	IT No.	Temporary No.	Resource Name	Origin[1]	Harvest time (d)	Leaf length (cm)	Shape	Leaf color	Inner color	Head weight (g)	Head height (cm)	Head width (cm)	Height-weight ratio	Core length (cm)
1	100498	**NA**	Bulamwolga	KOR	85	45	Flat	BRG	BRG	190	15.0	21.5	0.70	5.5
2	112268	**NA**	Busajosangamram	UNK	80	40	S-F	DG	BRG	267	18.5	21.8	0.85	6.5
3	136505	**NA**	Pride of India	NPL	65-70	27.5	Round	BRG	BRG	530	11.5	11.5	1.00	7.5
4	136507	**NA**	NPL-CGS-1986-22766	NPL	65-70	28	Round	DG	BRG	820	13.5	13.0	1.04	5.5
5	136509	**NA**	Drum Head	NPL	85	40	Flat	BRG	BRG	825	11.2	17.3	0.65	5.4
6	160330	**NA**	Jing Li 1	CHN	80-85	34	Flat	BRG	BRG	1070	12.0	20.0	0.60	5.5
7	160331	**NA**	Zhong gan 8	CHN	80-85	35	Flat	BRG	BRG	2020	14.0	25.0	0.56	5.0
8	160677	**NA**	Gyeongphong 1 ho	CHN	85	42	Flat	BRG	LG	1370	12.2	22.0	0.55	5.0
9	180791	**NA**	Late Flat Dutch	USA	90	38	Round	BRG	BRG	900	13.5	18.0	0.75	5.5
10	183701	**NA**	Copenhagen Market 86	ITA	80-85	31	Round	G	G	1830	17.0	16.5	1.03	7.0
11	189959	**NA**	Mozharskaya	RUS	85	42	Round	DG	LG	1275	14.3	16.8	0.85	4.7
12	189961	**NA**	Sud'ya	TKM	85	51	Round	G	LG	1370	14.8	19.2	0.77	4.8
13	189963	**NA**	Skvirskaya N32	UKR	90	44	Round	BRG	G	970	12.0	17.5	0.69	6.5
14	189965	**NA**	Zavadovskaya	KAZ	90	66	Round	G	G	1670	20.0	18.0	1.11	8.0
15	198909	**NA**	MNG-1996-3665	MNG	85	40	Round	DG	BRG	1205	15.7	16.8	0.93	7.2
16	203329	703048	Zavodovskaya	KAZ	90	40	Round	BRG	BRG	850	13.6	14.3	0.95	5.1
17	204203	707561	Golden Acre	USA	60-65	29	Round	BRG	LG	500	12.0	12.0	1.00	3.5
18	204207	707565	Green Express	USA	80-85	34.5	Round	G	G	2640	20.5	20.0	1.03	7.0
19	204220	707578	Slava	RUS	85	53	Round	BRG	BRG	1975	18.5	20.2	0.92	8.1
20	204326	709552	Nonkwawon-1998-3606	UNK	75-80	33	Round	BRG	G	1960	17.0	18.0	0.94	6.5
21	206819	711498	Kik-1998-6099	UNK	70-75	29	Round	G	G	940	16.0	12.0	1.33	14.5
22	**NA**	803346	Xymaiac	MNG	80-85	40	Semi-R	BRG	LG	1455	15.5	17.8	0.87	5.6
23	**NA**	803360	Zuun kharaa No 1	MNG	80-85	47	Round	BRG	LG	1540	14.7	17.8	0.83	7.1
24	**NA**	803361	Zuun kharaa No 2	MNG	80-85	40	Round	BRG, G	LG	1535	17.1	16.2	1.06	6.2
25	**NA**	803362	Zuun kharaa No 3	MNG	75-80	42	Round	BRG	LG	1570	18.0	17.0	1.06	8.0
26	**NA**	803363	Zuun kharaa No 4	MNG	80-85	45	Round	DG	BRG	1595	17.5	16.2	1.08	8.1
27	**NA**	803364	Zuun kharaa No 5	MNG	85-90	41	UP-R	BRG	LG	1385	15.9	14.7	1.08	5.2
28	**NA**	803365	Zuun kharaa No 6	MNG	85-90	48	Round	BRG	LG	1515	16.8	16.2	1.04	8.2
29	**NA**	803366	Zuun kharaa No 7	MNG	85	51	Round	BRG	LG	1630	17.5	16.2	1.08	6.8
30	**NA**	803367	Zuun kharaa No 8	MNG	85	42	Round	BRG	G,LG	1525	16.5	15.3	1.08	6.8
31	**NA**	803368	Zuun kharaa No 9	MNG	85	44	Round	BRG	BRG	1430	15.1	16.8	0.90	5.7
32	**NA**	803369	Zuun kharaa No 10	MNG	85	39	Round	BRG	G	1065	13.6	14.7	0.93	6.2
33	**NA**	803370	Zuun kharaa No 11	MNG	85	48	Round	BRG	BRG	1670	15.4	17.2	0.90	5.1
34	**NA**	803371	Zuun kharaa No 12	MNG	85	41	Round	BRG	BRG	1380	15.7	16.2	0.97	4.8
35	**NA**	803372	Zuun kharaa No 13	MNG	85	45	Round	BRG	G	1210	14.6	16.1	0.91	5.8
36	**NA**	803373	Zuun kharaa No 14	MNG	80-85	40	Round	BRG	BRG	1420	17.0	14.5	1.17	8.5
37	**NA**	803374	Zuun kharaa No 15	MNG	80-85	43	Round	BRG	G	1410	16.0	16.0	1.00	9.5
38	**NA**	803375	Zuun kharaa No 16	MNG	85-90	43	Round	BRG	G	1375	16.5	15.7	1.05	6.3
39	**NA**	803376	Zuun kharaa No 17	MNG	90	32	Round	G	G	600	14.5	12.5	1.16	5.5
40	**NA**	803377	Zuun kharaa No 18	MNG	85-90	50	Round	G	BRG	1610	17.1	18.2	0.94	6.8
41	**NA**	903575	NIS-1999-124	**NA***	70-75	38	Round	LG	LG	1350	14.5	15.5	0.94	6.0
42	**NA**	906760	Huyudori B-Go	JPN	95	23	Round	G	G	1000	14.0	18.0	0.78	7.0
43	**NA**	906762	Kinkei 201	**NA**	80	34	Round	DG	G	1460	16.0	16.0	1.00	6.5
44	**NA**	906763	Kenkei YR 21	**NA**	80	34	Round	DG	G	1460	16.0	16.0	1.00	6.5
45	**NA**	906764	Natsuzoka	**NA**	85	44	Round	BRG	G	1280	12.4	18.4	0.67	5.1
46	**NA**	906766	Ohzora	JPN	85	43	Round	BRG	G	1225	13.9	16.8	0.83	4.4
47	**NA**	906776	Super Red	**NA**	85	47	Round	P	P	520	10.5	11.7	0.90	5.6
48	**NA**	906777	Uji No 1	**NA**	85	46	Semi-F	G	G	1490	13.5	18.5	0.73	5.4
49	**NA**	906778	UR Gogetsu	**NA**	85	45	Semi-F	BRG	G	1015	14.2	16.1	0.88	4.5
50	**NA**	907279	Yujanka 31	RUS	85	45	Round	BRG	BRG	990	13.5	12.6	1.07	4.6
51	**NA**	908149	BOL-AWS-1999-153	**NA**	90	54	Flat	G,BRG	G,BRG	1160	10.0	17.5	0.57	5.5
52	**NA**	908150	BOL-AWS-1999-154	**NA**	75-80	32	Point	BRG	DG	1160	20.0	14.0	1.43	8.0
53	**NA**	908151	BOL-AWS-1999-156	**NA**	80	50	Round	BRG	BRG	610	15.0	19.5	0.77	5.5
54	**NA**	908154	BOL-AWS-1999-562	**NA**	85	44	Round	BRG	G	1510	15.2	18.4	0.83	6.4
55	**NA**	908775	Taskaram	UZB	85	55	Round	LG	LG	960	14.8	14.7	1.01	5.2
56	**NA**	908777	Beloko chanaya	UZB	85	55	Semi-F	LG	LG	1435	14.6	18.2	0.80	8.5
57	**NA**	K003963	THA-LSY-2000-76	THA	75-80	34	Point	BRG	G	1700	21.5	15.5	1.39	7.0
58	**NA**	K004353	Rubin	RUS	80-85	52	Round	PG	P	915	14.7	13.3	1.11	7.8
59	**NA**	K004354	Mars	RUS	80-85	34	Round	P	P	435	7.2	9.8	0.73	4.7
60	**NA**	K004495	WIR1855	**NA**	60-65	23	Round	BG	BG	440	11.0	11.0	1.00	4.0
61	**NA**	K004496	Zavadovskaya 257/263	RUS	60-65	32	Round	DG	G	920	17.0	13.5	1.26	5.0
62	**NA**	K004497	Derbentskaya Mestnaya Uluchshennaya 23	RUS	60-65	30	Round	G	G	910	17.0	16.0	1.06	6.0
63	**NA**	K004499	Iyunskaya 3200	RUS	75-80	36	Round	G	G	2800	19.2	19.8	0.97	9.8
64	**NA**	K004500	WIR 2590	**NA**	75-80	40	Semi-R	G	G	1990	17.0	20.0	0.85	9.0
65	**NA**	K004512	Luganskaya 7	UKR	75-80	36	R-P	BRG	G	2800	19.0	21.0	0.90	5.0
66	**NA**	K004515	Nomer Pervyipolyarnyi K-206	RUS	65-70	32.5	Round	BRG	LG	750	13.0	12.5	1.04	6.0
67	**NA**	K004520	Ditmarskaya	RUS	60-65	28	Round	LG	LG	860	13.0	14.0	0.93	6.0
68	**NA**	K004521	Belorusskaya 455	RUS	85-90	40	Round	BRG	G	1085	13.1	15.4	0.85	9.8
69	**NA**	K004523	Skorospelaya	RUS	60-65	27	Round	LG	LG	750	12.5	15.0	0.83	4.0
70	**NA**	K004524	Losinoostrovskaya	RUS	85-90	41	Round	BRG	G	1625	15.7	16.8	0.93	7.6
71	**NA**	K004525	Podarok	RUS	85-90	45	Semi-F	BRG	BRG	1040	12.4	19.6	0.63	5.3
72	**NA**	K004526	Sudiya-146	RUS	85-90	47	Semi-F	BRG	BRG	2265	17.2	23.3	0.74	10.4
73	**NA**	K004527	Kharkovskaya	UKR	85-90	44	Semi-F	BRG	BRG	895	14.3	15.4	0.93	7.8

Table S1: The source detail and agronomic characteristic of cabbage genotypes (n=146) (Cont^{n}.).

SN	IT No.	Temporary No.	Resource Name	Origin[1]	Harvest time (d)	Leaf length (cm)	Shape	Leaf color	Inner color	Head weight (g)	Head height (cm)	Head width (cm)	Height-weight ratio	Core length (cm)
74	**NA**	K004532	Moskovskaya Pozdnyaya	RUS	85-90	52	Round	LG	LG	1525	19.1	17.8	1.07	6.6
75	**NA**	K004535	Slava 1305	RUS	85	45	Round	G	G	1935	18.2	17.4	1.05	7.8
76	**NA**	K004536	Nadezhda	RUS	85	53	Semi-F,P	LG	G	1255	14.9	17.1	0.87	6.1
77	**NA**	K004538	Iyunskaya	RUS	65-70	30	Round	LG	LG	1210	16.5	13.5	1.22	8.0
78	**NA**	K004540	Zinovka 1447	RUS	85	53	Round	LG	LG	925	17.5	15.7	1.11	8.3
79	**NA**	K004542	Slava 1305	RUS	80	52	Round	BRG	G	1105	15.3	14.8	1.03	6.5
80	**NA**	K004543	Polyarnaya 206	RUS	65-70	32	Round	BRG	G	1070	16.0	14.0	1.14	6.0
81	**NA**	K004545	Nomer Pervyi Gribovskii 47	RUS	65-70	33	Round	G	G	980	16.0	13.0	1.23	4.0
82	**NA**	K004546	Moskovskaya Pozdnyaya	RUS	80	43	Round	BRG	BRG	1020	16.2	15.3	1.06	5.0
83	**NA**	K005289	Dregonbul	**NA**	65-70	31	Round	G	G	1500	15.0	16.0	0.94	6.5
84	**NA**	K005290	Janggun	**NA**	80	43	Semi-F	G	G	2070	14.7	20.6	0.71	8.8
85	**NA**	K014433	No.1	MNG	80	39	Round	G	G	800	13.6	12.8	1.06	6.5
86	**NA**	K014950	CHN-MKH-2001-100	**NA**	65-70	29	Round	G	G	1810	16.5	16.5	1.00	5.0
87	**NA**	K014955	CHN-MKH-2001-139	**NA**	65-70	29	Round	G	G	1300	15.0	15.5	0.97	6.0
88	**NA**	K044570	NPL-GYS-2004-26	NPL	65-70	33	Round	BRG	BRG	710	12.5	13.0	0.96	4.5
89	**NA**	K045062	Kashirka 202	RUS	80	48	Round	BRG	BRG	790	13.3	14.4	0.92	6.5
90	**NA**	K045064	Valcatiecskaya	RUS	80	42	Round	BRG	BRG	920	14.1	13.5	1.04	3.8
91	**NA**	K060636	roxo	**NA**	80	41	Round	GP	P	695	14.3	11.2	1.28	6.3
92	**NA**	K100196	Predzvest	**NA**	65-70	28	Round	G	G	1280	18.0	17.0	1.06	8.5
93	**NA**	K100197	Pourovo cervene	**NA**	80	53	Round	GP	P	960	15.7	12.8	1.23	6.4
94	**NA**	K121377	UZB-KJG-2006-37	KGZ	80	46	Round	BRG	LG	830	12.8	14.2	0.90	8.3
95	**NA**	K125498	Paz. Podobr	**NA**	80	50	Round	GP	GP	1080	14.4	16.5	0.87	5.8
96	**NA**	K125499	Kose 27	**NA**	80	43	Round	DG	LG	1285	15.1	16.4	0.92	9.1
97	**NA**	K134135	AT16	TUR	80	43	Round	GP	P	1070	14.7	13.1	1.12	5.1
98	**NA**	K134260	HA15	TUR	90	48	Round	GP	P	720	12.5	11.0	1.14	8.5
99	**NA**	K134820	Pazardzhishko podobreno 16	BGR	80	50	Round	LP	LP	1140	14.5	16.2	0.90	6.1
100	**NA**	K136870	Kyuse (=bold)	BGR	90	56	Flat	BRG	BRG	1750	15.0	19.5	0.77	8.5
101	**NA**	K138940	C.M	**NA**	65-70	30	Round	BRG	BRG	1130	15.0	15.0	1.00	7.0
102	**NA**	K139046	Rethudoru-Khi	**NA**	80	38	Round	GP	P	1180	12.4	15.7	0.79	6.8
103	**NA**	K139130	Sagyahwak	**NA**	85	57	Round	G	G	1205	14.3	17.8	0.80	4.2
104	**NA**	K139318	Okhina	**NA**	75-80	34	Semi-F	BRG	G	2730	17.5	22.0	0.80	6.0
105	**NA**	K142931	Sudya	KGZ	90	32	Round	BRG	BRG	890	13.0	16.0	0.81	8.0
106	**NA**	K145099	Kantarotopu	TUR	85	38	Round	BRG	P	950	15.3	12.7	1.20	6.1
107	**NA**	K145187	Kirmizi	TUR	90	32	Round	GP	P	830	13.0	10.0	1.30	7.0
108	**NA**	K146556	A8E0243	BGR	85	46	Round	BRG	G	1715	17.1	17.4	0.98	5.5
109	**NA**	K154726	Akimakigokuwase	JPN	75-80	37	R, P	G	LG	1430	16.0	20.0	0.80	7.5
110	**NA**	K155038	Tashkent 110	UZB	85	47	Round	DG	LG	925	13.2	14.8	0.89	6.6
111	**NA**	K155067	UZB-GJG-2009-10/3-09	**NA**	85	46	Round	LG	LG	1590	15.4	19.3	0.80	9.2
112	**NA**	K155659	CT-99	KOR	65-70	29	Round	G	G	1060	14.0	16.0	0.88	6.0
113	**NA**	K155662	CT-115	KOR	75-80	34	Round	DG	G	1300	16.0	14.0	1.14	5.0
114	**NA**	K155664	CT-117	KOR	80	40	Round	DG	G	1545	16.6	15.2	1.09	5.7
115	**NA**	K164144	CHN-AWS-2010-12	CHN	65-70	26	Round	G	G	1090	14.5	14.0	1.04	3.5
116	**NA**	K164742	Charleston wakefield	PER	75-80	37	R, P	G	G	1210	17.0	15.5	1.10	6.5
117	**NA**	K166200	1740	KOR	85	42	Round	BRG	BRG	475	9.2	11.3	0.81	4.2
118	**NA**	K166201	1780	KOR	75-80	38	Round	BRG	BRG	1380	14.0	17.0	0.82	5.0
119	**NA**	K166205	153	KOR	100	41.5	Round	DG	G	1000	15.0	14.0	1.07	6.0
120	**NA**	K166231	Ne 1	CHN	60-65	31	Round	G	G	1290	15.0	17.0	0.88	5.0
121	**NA**	K166237	Doberrot	DEU	85	33	Round	P-G	P	670	13.1	10.2	1.28	5.3
122	**NA**	K166240	Red Drumhead 2	SWE	85	43	Round	P-G	P-G	490	12.5	10.2	1.23	4.4
123	**NA**	K175584	213	KOR	65-70	33.5	Round	G	G	1260	15.5	14.0	1.11	5.5
124	**NA**	K176590	Da shanghai xin zheng chin ganlan	CHN	75-80	41	Round	G	G	2160	20.0	17.0	1.18	5.0
125	**NA**	K176592	Qing Feng	CHN	80-85	40	Round	BRG	BRG	2330	19.0	19.5	0.97	5.5
126	**NA**	K176593	Zhong Gan 21	CHN	60-65	29	Round	G	G	1240	16.5	17.0	0.97	5.5
127	**NA**	K176594	Zao Hong	CHN	75-80	30	Round	P	P	2430	18.0	17.5	1.03	12.0
128	**NA**	K176595	Zhong Gan 11	CHN	65-70	32	Round	G	G	1250	15.0	16.0	0.94	6.0
129	**NA**	K195951	Golden Acre Imp	IND	65-70	30	Round	G	G	710	12.0	14.0	0.86	4.0
130	**NA**	K204466	CT-260	KOR	65-70	22	Round	G	G	750	12.5	12.0	1.04	4.0
131	**NA**	K222934	CH 83	KOR	80-85	32	Round	BRG	BRG	980	14.5	13.1	1.11	6.1
132	**NA**	K229554	PER-LYH-2013-3	PER	75-80	32	Point	BL	BL	1232	20.0	15.0	1.33	10.0
133	**NA**	K243791	KS 142	THA	80	41	Round	PG	P	790	13.5	13.0	1.04	6.5
134	**NA**	K243819	Miya Bi	THA	80-85	42	Round	G	G	1865	14.4	19.2	0.75	8.1
135	**NA**	K246353	90ms	KOR	80-85	34	Round	BRG	BRG	1140	15.8	13.6	1.16	7.2
136	**NA**	K246359	P15-41	KOR	80-85	30	Round	G	G	1265	14.5	15.7	0.92	5.4
137	**NA**	K246894	Succession Green Leaved	ARM	80-85	45	Flat	BRG	BRG	750	10.3	16.8	0.61	6.4
138	**NA**	K246912	A5-7	TWN	75-80	46	Round	BRG	G	1380	14.0	16.0	0.88	7.0
139	**NA**	K247130	N 127	**NA**	95	39	Round	G	G	830	14.0	13.5	1.04	6.0
140	**NA**	K247132	Mihnevskaya	RUS	80-85	34	Round	PG	PG	1750	14.2	13.1	1.08	6.7
141	**NA**	K247133	Kopengagenskaya	**NA**	80-85	50	Round	PG	PG	1160	13.7	13.8	0.99	9.5
142	**NA**	K247714	TJK-PHJ-2014-2-21	TJK	80-85	47	Round	BRG	G	1410	15.7	16.4	0.96	9.3
143	**NA**	K247741	TJK-PHJ-2014-6-8	TJK	80-85	43	Round	BRG	BRG	1125	14.9	15.6	0.96	5.2
144	**NA**	K247748	TJK-PHJ-2014-6-16	UZB	80-85	48	Round	BRG	G	1345	14.6	14.2	1.03	6.2
145	**NA**	K247794	Apsheronskaya	TJK	80-85	47	Round	G	G	910	17.2	13.8	1.25	4.2
146	**NA**	K254252	Likani	GEO	80-85	40	Round	G	G	795	15.8	15.2	1.04	5.2

NA: information not available; R,P: round and pointed; Semi-F: Semi-flat;; Semi-R: semi-round; UP-R: upper pointed-round; BRG: bright green; DG: dark green; G: green; LG: light green; P-G: purple green, P: purple. Origin[1]: α-3 country code.

Table S2: Variation in GSLs profile and concentration (μmol g^{-1} DW) in cabbage head (n=146).

SN	IT No.	Temporary No.	GIB	PRO	EPI	SIN	GRA	GNA	4HGBS	GER	GBS	4MGBS	GNS	NGBS	Total GSLs
1	100498	NA	0.75	0.25	0.12	1.87	0.04	0.15	0.08	ND	3.74	0.33	0.05	0.32	7.70
2	112268	NA	1.21	0.38	0.06	1.07	0.20	0.14	0.13	ND	5.72	0.86	ND	0.11	9.87
3	136505	NA	3.01	0.60	0.11	7.22	0.22	0.57	0.03	ND	4.72	0.23	ND	0.64	17.33
4	136507	NA	3.68	0.67	0.12	6.92	0.35	0.36	0.05	ND	4.80	0.12	0.24	0.33	17.65
5	136509	NA	2.59	0.62	0.15	6.45	0.22	0.27	0.04	ND	6.00	0.18	ND	0.93	17.44
6	160330	NA	3.20	0.22	0.08	2.64	0.13	0.14	0.02	ND	3.77	0.36	ND	0.16	10.72
7	160331	NA	0.57	2.29	0.17	1.24	0.83	0.65	ND	ND	5.96	0.15	0.12	0.28	12.27
8	160677	NA	3.15	0.86	0.10	6.33	0.31	0.25	0.17	ND	4.50	0.75	0.62	0.14	17.17
9	180791	NA	4.87	0.49	0.09	5.77	0.31	0.23	0.11	ND	9.08	0.27	0.12	1.27	22.60
10	183701	NA	0.86	0.36	0.10	7.33	ND	0.16	0.04	ND	2.36	0.49	ND	0.43	12.13
11	189959	NA	0.80	1.67	0.14	0.70	2.41	0.73	0.05	ND	8.67	0.43	0.16	1.57	17.32
12	189961	NA	0.05	ND	0.11	0.09	2.06	ND	0.05	ND	4.50	0.28	0.16	0.94	8.26
13	189963	NA	2.02	0.45	0.14	2.98	0.22	0.22	0.10	ND	10.10	0.38	ND	0.20	16.81
14	189965	NA	0.55	0.70	0.11	0.16	2.60	0.41	0.16	ND	5.54	0.38	ND	0.46	11.07
15	198909	NA	1.52	0.38	0.12	3.70	0.12	0.32	0.07	ND	2.95	0.19	0.08	0.51	9.96
16	203329	703048	2.35	0.60	0.13	5.55	0.22	0.29	ND	ND	6.94	0.19	0.19	0.16	16.62
17	204203	707561	2.10	0.32	0.09	3.28	0.05	0.07	0.01	ND	3.48	0.10	0.10	1.70	11.32
18	204207	707565	0.89	0.45	0.09	6.07	0.06	0.41	0.07	ND	1.32	0.28	0.23	0.20	10.07
19	204220	707578	2.60	0.33	0.11	2.51	0.35	0.27	0.05	ND	4.92	0.17	0.15	0.44	11.90
20	204326	709552	1.37	0.21	0.13	3.22	0.06	0.25	0.04	ND	2.25	0.11	0.19	0.46	8.29
21	206819	711498	1.82	0.39	0.18	5.72	0.08	0.18	0.02	ND	2.42	0.15	0.14	0.21	11.31
22	NA	803346	3.12	0.04	0.08	2.09	0.06	0.06	0.02	ND	1.74	0.29	ND	0.20	7.70
23	NA	803360	4.06	0.20	0.08	4.48	0.11	0.13	0.03	ND	4.03	0.19	ND	0.25	13.55
24	NA	803361	0.62	0.64	0.11	0.77	0.70	0.46	0.03	ND	2.67	0.21	ND	0.20	6.40
25	NA	803362	0.61	0.61	0.14	0.93	0.43	0.45	0.07	ND	2.37	0.14	0.07	0.18	5.99
26	NA	803363	3.13	0.16	0.10	3.88	0.13	0.11	0.04	ND	4.42	0.21	0.13	0.75	13.05
27	NA	803364	2.25	0.11	0.07	4.00	0.07	0.12	0.21	ND	2.35	0.54	0.14	0.68	10.53
28	NA	803365	1.32	0.52	0.14	7.22	0.20	0.35	0.03	ND	2.18	0.20	0.30	0.17	12.62
29	NA	803366	1.00	1.90	0.14	2.10	1.27	1.77	0.16	0.15	2.83	0.16	0.07	0.11	11.65
30	NA	803367	1.48	0.33	0.09	4.44	0.20	0.27	0.04	ND	1.07	0.21	0.11	0.12	8.36
31	NA	803368	0.73	0.10	0.08	3.18	0.03	0.10	0.05	ND	3.79	0.20	0.09	0.97	9.32
32	NA	803369	2.35	0.52	0.10	10.29	0.13	0.56	0.04	ND	4.38	0.17	0.28	0.56	19.38
33	NA	803370	0.30	2.15	0.22	1.53	0.45	2.24	0.05	ND	0.79	0.12	0.07	0.11	8.02
34	NA	803371	1.72	0.83	0.13	1.32	1.26	0.50	0.04	0.10	2.67	0.13	0.09	0.13	8.92
35	NA	803372	2.20	0.14	0.12	1.46	0.18	0.14	0.04	ND	1.50	0.20	ND	0.29	6.28
36	NA	803373	2.13	0.26	0.10	2.97	0.20	0.15	0.07	ND	7.40	0.27	0.31	0.39	14.24
37	NA	803374	4.55	0.50	0.10	8.28	0.21	0.32	0.04	ND	4.51	0.40	ND	0.30	19.21
38	NA	803375	0.89	1.69	0.14	1.12	1.56	1.21	0.05	ND	2.82	0.30	ND	0.35	10.13
39	NA	803376	1.38	0.34	0.06	5.09	0.06	0.15	0.07	ND	2.42	0.07	0.08	0.34	10.07
40	NA	803377	2.52	0.30	0.11	4.51	0.19	0.32	0.17	ND	4.10	0.14	0.23	0.50	13.07
41	NA	903575	2.22	0.46	0.08	7.88	0.10	0.35	ND	ND	3.00	0.13	0.47	0.29	14.99
42	NA	906760	1.47	0.52	ND	4.86	0.18	0.24	0.02	ND	3.50	0.36	0.16	0.36	11.66
43	NA	906762	0.57	0.20	0.09	0.83	0.09	0.13	0.03	0.10	2.93	0.29	ND	0.10	5.36
44	NA	906763	0.78	0.19	0.07	0.99	0.10	0.14	0.17	0.11	2.52	0.50	ND	0.08	5.64
45	NA	906764	1.08	3.28	0.22	2.17	1.47	1.60	0.08	0.12	7.46	0.13	ND	0.22	17.82
46	NA	906766	1.76	0.34	0.16	3.91	0.13	0.13	0.06	ND	6.32	0.18	0.12	0.15	13.26
47	NA	906775	0.91	1.72	0.14	1.90	0.66	0.88	0.10	ND	6.60	1.01	ND	0.43	14.35
48	NA	906776	1.64	0.11	0.13	4.47	0.10	0.07	0.02	ND	4.34	0.18	ND	0.23	11.29
49	NA	906777	2.11	0.44	0.11	2.81	0.29	0.23	0.02	ND	10.60	0.47	0.26	0.32	17.65
50	NA	907279	2.79	1.03	0.09	11.29	0.20	0.53	0.06	ND	3.25	0.10	0.24	0.28	19.86
51	NA	908149	2.92	1.17	0.12	12.87	0.20	0.61	0.11	ND	4.09	0.28	0.29	0.74	23.39
52	NA	908150	2.20	0.57	0.13	4.00	0.19	0.29	0.08	ND	4.45	0.25	ND	0.33	12.49
53	NA	908151	3.86	0.20	0.11	3.46	0.20	0.16	0.03	ND	13.14	0.21	0.67	1.73	23.75
54	NA	908154	0.87	2.38	0.19	2.80	1.05	1.88	0.04	ND	6.12	0.08	0.13	0.32	15.86
55	NA	908775	0.85	ND	0.15	ND	1.64	0.03	0.02	ND	4.36	0.24	ND	0.10	7.40
56	NA	908777	0.04	ND	0.12	ND	1.81	0.01	ND	ND	5.18	0.21	ND	0.10	7.48
57	NA	K003963	0.31	0.42	0.09	2.94	0.04	0.22	0.04	ND	4.34	0.29	ND	0.12	8.83
58	NA	K004353	1.00	3.28	0.17	2.56	2.04	ND	0.15	ND	7.76	0.19	ND	0.40	17.55
59	NA	K004354	0.38	1.21	0.09	0.66	1.27	ND	0.06	ND	5.20	0.33	ND	0.62	9.83
60	NA	K004495	1.92	0.60	0.08	3.62	0.14	0.14	0.03	ND	2.48	0.19	0.16	1.18	10.53
61	NA	K004496	1.56	0.87	0.10	2.50	1.09	0.41	0.03	ND	2.81	0.16	0.31	0.66	10.51
62	NA	K004497	1.77	0.20	0.06	2.91	0.04	0.08	0.02	ND	1.07	0.19	0.07	0.73	7.15
63	NA	K004499	1.76	0.29	0.12	5.18	0.18	0.27	0.06	ND	2.88	0.25	0.18	0.27	11.44
64	NA	K004500	1.32	0.28	0.10	3.51	0.05	0.16	0.12	ND	3.63	0.65	0.10	1.09	11.02
65	NA	K004512	1.58	0.23	0.08	2.42	0.10	0.12	0.18	ND	1.34	0.36	0.11	0.23	6.74
66	NA	K004515	2.64	0.26	0.15	1.71	0.39	0.09	0.09	ND	8.97	0.60	0.15	0.87	15.92
67	NA	K004520	1.11	0.31	0.06	5.61	0.05	0.14	0.05	ND	1.99	0.26	0.15	0.64	10.36
68	NA	K004521	2.05	0.47	0.17	3.64	0.32	0.58	0.06	ND	3.23	0.10	ND	0.26	10.86
69	NA	K004523	0.85	0.36	0.09	3.29	0.03	0.13	0.07	ND	1.28	0.28	0.09	0.36	6.83
70	NA	K004524	0.80	1.60	0.13	2.19	0.71	1.73	0.09	ND	4.05	0.14	0.13	0.45	12.03
71	NA	K004525	5.01	0.53	0.11	5.67	0.35	0.30	0.29	ND	5.87	0.37	0.16	0.80	19.48
72	NA	K004526	0.05	1.62	0.11	0.03	3.85	0.52	0.07	0.18	2.51	1.07	0.22	0.15	10.39
73	NA	K004527	2.90	0.16	0.14	2.47	0.14	0.12	0.03	ND	2.55	0.09	0.07	0.18	8.84

Table S2: Variation in glucosinolate profile and concentration (µmol g^{-1} DW) in cabbage head (n=146) (Cont^{n}.)

SN	IT No.	Temporary No.	GIB	PRO	EPI	SIN	GRA	GNA	4HGBS	GER	GBS	4MGBS	GNS	NGBS	Total GSLs
74	NA	K004532	2.70	0.14	0.14	2.08	0.15	0.10	0.04	ND	2.76	0.43	0.08	0.18	8.80
75	NA	K004535	0.90	2.72	0.25	4.31	0.58	2.05	0.08	ND	1.57	0.24	0.14	0.15	12.97
76	NA	K004536	2.62	1.13	0.18	8.15	0.51	0.45	0.10	ND	4.11	0.13	0.14	0.62	18.13
77	NA	K004538	1.12	0.26	0.06	4.33	0.05	0.19	0.05	ND	3.04	0.35	0.14	0.58	10.17
78	NA	K004540	1.71	0.31	0.14	3.61	0.16	0.22	0.11	ND	6.97	0.20	0.11	0.17	13.71
79	NA	K004542	1.90	0.31	0.12	3.86	0.25	0.28	0.05	ND	2.89	0.16	ND	0.17	9.99
80	NA	K004543	1.48	2.25	0.15	5.79	0.66	1.50	0.03	ND	2.38	0.15	0.23	0.37	14.98
81	NA	K004545	1.96	0.48	0.09	6.25	0.09	0.27	0.08	ND	3.30	0.22	0.14	0.36	13.23
82	NA	K004546	0.15	0.69	0.14	8.26	ND	0.80	0.18	ND	3.71	0.26	ND	0.14	14.32
83	NA	K005289	1.12	0.23	0.07	4.26	0.03	0.16	0.11	ND	1.42	0.25	0.14	0.06	7.87
84	NA	K005290	0.93	0.19	0.09	1.23	0.08	0.07	0.03	ND	3.00	0.11	0.09	0.19	6.01
85	NA	K014433	2.79	0.70	0.08	7.61	0.30	0.39	0.12	0.12	4.71	0.22	ND	0.49	17.53
86	NA	K014950	2.04	0.75	0.07	4.69	0.27	ND	0.04	ND	1.44	0.23	ND	0.46	9.99
87	NA	K014955	1.38	0.43	0.10	3.58	0.10	ND	0.04	ND	2.48	0.24	ND	0.22	8.57
88	NA	K044570	1.74	0.36	0.08	6.73	0.05	0.29	0.02	ND	1.76	0.09	0.10	0.31	11.54
89	NA	K045062	3.16	0.90	0.12	6.88	0.54	0.46	0.28	ND	7.97	0.29	0.12	0.90	21.63
90	NA	K045064	1.22	2.06	0.21	10.74	0.30	1.93	0.04	0.15	5.88	0.21	ND	0.60	23.33
91	NA	K060636	0.74	1.97	0.15	0.94	2.64	ND	0.06	ND	3.96	ND	ND	0.44	10.90
92	NA	K100196	0.86	0.08	0.06	3.92	ND	ND	ND	ND	3.98	0.19	ND	0.41	9.50
93	NA	K100197	0.31	2.49	0.18	0.77	1.47	2.81	0.07	0.10	3.26	0.16	ND	0.44	12.05
94	NA	K121377	1.25	0.02	0.11	0.02	2.23	0.06	0.05	0.15	6.80	0.32	ND	0.75	11.76
95	NA	K125498	0.04	ND	0.08	ND	1.23	ND	0.08	ND	7.62	0.16	ND	0.26	9.48
96	NA	K125499	0.28	ND	0.07	0.03	0.90	0.03	0.05	ND	1.75	0.59	ND	0.28	3.99
97	NA	K134135	0.70	1.04	0.11	0.92	1.11	0.68	0.07	0.13	3.38	0.10	ND	0.20	8.45
98	NA	K134260	0.98	1.82	0.09	2.33	0.73	1.37	0.09	0.13	2.04	0.12	ND	0.12	9.82
99	NA	K134820	0.01	0.02	0.10	ND	2.31	0.11	0.03	0.21	5.76	0.78	ND	0.40	9.73
100	NA	K136870	0.30	1.06	0.10	0.23	0.75	0.27	0.09	ND	3.40	0.43	0.13	0.87	7.63
101	NA	K138940	2.10	0.27	0.09	3.40	0.12	0.11	0.05	ND	1.69	0.21	0.15	0.26	8.44
102	NA	K139046	0.20	1.70	0.17	0.42	0.70	1.21	0.11	ND	3.15	0.72	ND	0.40	8.78
103	NA	K139130	3.39	0.89	0.15	10.05	0.26	0.49	0.09	ND	4.90	0.17	0.24	0.10	20.72
104	NA	K139318	0.78	2.04	0.20	1.94	0.74	1.45	0.06	0.10	3.18	0.32	0.09	0.08	10.91
105	NA	K142931	0.01	0.01	0.04	0.01	3.36	0.03	0.06	0.26	4.03	2.57	ND	0.39	10.77
106	NA	K145099	0.33	1.20	0.11	0.60	0.69	0.91	0.05	ND	3.92	ND	ND	0.48	8.29
107	NA	K145187	0.57	3.41	0.15	2.00	1.53	2.79	ND	0.16	6.40	0.22	ND	0.96	18.19
108	NA	K146556	0.27	0.02	0.07	0.01	1.03	0.02	0.03	ND	2.30	0.24	ND	0.08	4.05
109	NA	K154726	1.28	0.60	0.07	1.71	0.34	0.32	0.06	0.12	2.87	0.25	ND	0.15	7.77
110	NA	K155038	0.05	3.86	0.26	0.09	2.65	0.92	0.04	0.56	5.47	0.45	ND	0.35	14.69
111	NA	K155067	0.64	ND	0.14	0.05	2.15	ND	ND	0.31	5.59	0.67	0.21	0.19	9.95
112	NA	K155659	1.36	0.41	0.08	4.59	0.05	0.22	0.03	ND	1.74	0.26	0.07	0.26	9.05
113	NA	K155662	1.36	0.62	0.11	8.17	0.26	1.03	0.02	0.14	3.52	0.22	0.12	0.28	15.85
114	NA	K155664	1.48	0.34	0.12	5.69	0.11	0.26	0.02	ND	2.23	0.24	ND	0.48	10.97
115	NA	K164144	0.43	0.02	0.10	0.08	2.33	0.03	0.03	0.28	4.27	0.21	ND	0.52	8.29
116	NA	K164742	0.42	2.32	0.17	1.45	0.50	2.11	0.03	0.18	2.39	0.10	0.06	0.22	9.94
117	NA	K166200	2.46	0.88	0.18	3.79	0.55	0.32	ND	ND	4.81	0.28	ND	0.34	13.60
118	NA	K166201	0.80	1.40	0.14	1.36	0.59	0.49	0.06	ND	3.08	0.11	0.12	0.08	8.23
119	NA	K166205	1.84	0.08	0.09	4.11	0.06	0.08	0.02	ND	6.34	0.22	ND	2.00	14.83
120	NA	K166231	2.11	0.22	0.08	3.44	0.09	0.08	0.08	ND	1.68	0.38	0.08	0.35	8.60
121	NA	K166237	0.51	1.73	0.16	0.71	1.63	1.16	0.06	ND	3.03	0.08	ND	0.34	9.41
122	NA	K166240	0.65	3.06	0.19	1.49	1.78	2.36	0.06	0.22	4.42	ND	ND	0.16	14.41
123	NA	K175584	1.25	0.61	0.06	6.03	ND	0.50	0.01	ND	2.74	0.27	ND	0.21	11.70
124	NA	K176590	0.44	0.27	0.05	1.27	0.03	0.14	0.04	ND	1.89	0.22	ND	0.14	4.51
125	NA	K176592	2.12	0.34	0.06	4.22	0.18	0.23	0.06	ND	1.45	0.22	ND	0.22	9.09
126	NA	K176593	0.69	2.40	0.16	1.65	0.53	1.20	0.03	ND	1.32	0.26	0.07	0.20	8.50
127	NA	K176594	0.30	1.57	0.17	0.58	1.54	1.08	0.04	ND	4.38	0.17	ND	0.21	10.04
128	NA	K176595	1.34	0.19	0.06	3.62	ND	0.09	ND	ND	1.90	0.14	ND	0.27	7.62
129	NA	K195951	2.86	0.33	0.08	6.66	0.08	0.14	0.10	ND	2.57	0.33	ND	0.56	13.72
130	NA	K204466	2.60	0.40	0.07	5.26	0.08	0.31	0.01	ND	3.78	0.34	ND	0.49	13.34
131	NA	K222934	0.80	0.06	0.08	2.30	ND	0.04	0.03	ND	2.18	0.20	ND	0.31	6.01
132	NA	K229554	1.29	0.48	0.08	3.33	0.17	0.34	0.03	ND	2.54	0.14	ND	0.11	8.49
133	NA	K243791	0.41	1.73	0.11	0.90	1.44	2.11	ND	ND	4.50	0.12	ND	0.40	11.71
134	NA	K243819	1.39	1.40	0.11	5.50	0.44	0.56	0.03	ND	2.14	0.11	0.36	0.05	12.08
135	NA	K246353	1.30	0.26	0.12	4.00	0.05	0.12	0.02	ND	2.02	0.09	ND	0.10	8.06
136	NA	K246359	1.24	0.06	0.09	1.67	0.01	0.03	0.03	ND	3.24	0.43	ND	0.34	7.12
137	NA	K246894	2.83	0.25	0.14	9.53	0.09	0.05	0.06	ND	7.48	0.41	ND	0.49	21.34
138	NA	K246912	2.16	0.28	0.12	2.26	0.13	0.07	0.02	ND	4.98	0.34	ND	0.37	10.72
139	NA	K247130	1.44	0.56	0.10	8.19	0.13	0.29	0.04	ND	6.05	0.22	ND	0.67	17.69
140	NA	K247132	0.40	0.91	0.09	0.86	0.47	0.60	ND	ND	3.64	0.22	ND	0.21	7.38
141	NA	K247133	1.82	0.32	0.10	5.60	ND	0.28	0.02	ND	2.58	0.07	0.44	0.08	11.31
142	NA	K247714	0.05	0.04	0.09	0.06	2.77	0.03	0.03	0.61	2.72	0.53	ND	0.47	7.41
143	NA	K247741	0.78	0.04	0.09	0.02	3.10	0.04	0.04	ND	3.72	0.69	0.27	0.11	8.90
144	NA	K247748	1.35	1.08	0.12	2.59	1.13	0.53	0.07	ND	3.09	0.61	0.36	0.13	11.07
145	NA	K247794	0.56	1.52	0.16	0.58	1.43	0.76	0.06	ND	3.79	0.22	0.56	0.28	9.92
146	NA	K254252	2.10	0.54	0.10	5.06	0.14	0.26	ND	ND	2.72	1.41	ND	0.54	12.86

Each value represents the average of 3 replications. NA: Information not available; ND: Not detected. GIB: glucoiberin; PRO: progoitrin; EPI: epiprogoitrin; SIN: sinigrin; GRA: glucoraphanin; GNA: gluconapin; GER: glucoerucin; 4HGBS:4-hydroxyglucobrassicin; GBS: glucobrassicin; 4MGBS: 4-methoxyglucobrassicin; NGBS: neoglucobrassicin; GNS: gluconastrutiin.
